# Enhanced Bandwidth of High Directive Emission Fabry-Perot Resonator Antenna with Tapered Near-Zero Effective Index Using Metasurface

**DOI:** 10.1038/s41598-017-11141-z

**Published:** 2017-09-13

**Authors:** Zhen-Guo Liu, Wei-Bing Lu, Wu Yang

**Affiliations:** 1 State Key Laboratory of Millimeter Waves, Southeast University, Nanjing, 210096 China; 2 School of Information Science and Engineering, Southeast University, Nanjing, 210096 China; 3 School of Computer Science and Engineering, Southeast University, Nanjing, 210096 China

## Abstract

In this paper, a novel explanation on high directive emission of Fabry-Perot resonator antenna with subwavelength metasurface is proposed. Based on image theory and effective constitutive parameter retrieval, the whole Fabry-Perot resonant cavity structure composed of a single-layer metasurface with square ring element and a PEC ground plate can be acted as an effective metamaterial media with very low refractive index (near zero index). According to Snell’s theory, this property can be used to enhance the directive emission. Based on this, with tapered size square ring unitcell, the overlapped bandwidth in which the effective refractive index is near to zero is obtained to widen the bandwidth of high directive emission. It is demonstrated that the maximum of directivity is nearly approaching to 19 dBi, and its 3-dB bandwidth can be improved to 19.5%. A final prototype has been fabricated and measured to validate the proposed design concept. The measured 3-dB gain bandwidth is approximately 20.3% with a peak gain of 17.9 dBi. These results indicate the feasibility of such kind of antenna for broadband and high directivity applications simultaneously.

## Introduction

Fabry-Perot resonator antenna (FPRA), with high directive emission characteristic, generally consists of a primary radiator backed with a metal ground plate and a partially reflective covered plateor partial reflective surface^1^ (PRS), has also proposed for several years. Their highly directional emission properties and low complexity have aroused more and more attention^2–5^. But its inherent shortcoming is obviously narrow fractional bandwidth of directive emission, which is limited its applications in some communication system. In addition, a slab lens with gradient refractive index metamaterial has also been designed to locate on the aperture of horn antenna in order to enhance its directivity^6–9^. Although, these two type of antennas mentioned above both have high directivity properties, there are obviously differences between them not only in structure but also in mechanism of high directive emission. For horn antenna with gradient refractive index metamaterial slab cover^6–8^, the distance between primary source (generally an inner conductor of coaxial line protrude into the waveguide with shorted end) and the slab cover is around 3–4 or more wavelength level, thus the effect of slab cover on primary source or mutual coupling between them is generally very small that can be ignored. It means that the properties of metamaterial slab cover can be retrieved independently. So high directivity characteristics of this type antenna can be explained by lens with gradient refractive index. But for FPRA, due to the distance between the metal ground plate and the PRS is generally in half wavelength level or less, and the forward radiation in the aperture of antenna can be enhanced remarkably by means of in-phase bouncing between them. It implies that the interaction and mutual coupling between the metal ground plate and the PRS is larger enough that must be considered seriously. So the constitution of PRS in FPRA should not be retrieved independently.

On the other hand, recently, planar metasurfaces^10–12^ composed of subwavelength elements arranged into a sheet of subwavelength thickness have been attracting considerable attention for their demonstrated ability to efficiently manipulating the electromagnetic wavefront^13–18^. Compared with three dimension metamaterial, metasurface due to its two dimensional geometry inherently leads to low-profile devices with reduced losses and far less complex design, making them very attractive for many applications. As different from volumetric metamaterials, which are usually characterized by effective permittivity and permeability, metasurfaces have been characterized by its surface parameters, in terms of surface polarizabilities, surface susceptibilities, surface impedances, or surface admittances^10–19^. Hence, in principle, impinged by plane wave, a desirable EM-field distribution in space can be achieved by engineering and designing the surface to induce currents that would produce the required tangential fields on both of its facets. But, the procedure of analysis and design metasurface above also imply that the distance between source and metasurface sheet must be large enough to ignore the mutual coupling between them. Thus in this work although the metasurface is used as superstrate of FPR to enhance antenna boresight emission, based on above investigation, the general analysis approach to metasurface such as generalized sheet transition condition (GSTC) method^10–12^ is not suitable for analyzing the metasurface involved in FPRA.

Therefore, in this paper, a novel analysis approach to metasurface used as PRS in FPRA is proposed. Based on image theory, under the consideration of mutual coupling, the whole structure of FP cavity composed of metasurface superstrate and ground plate can be acted as an effective metamaterial media^5^. Furthermore, based on this viewpoint, a broadband FPRA with high directive emission property is also designed and presented.

## Results

### Constitutive Parameters Retrieval

**Figure 1:**
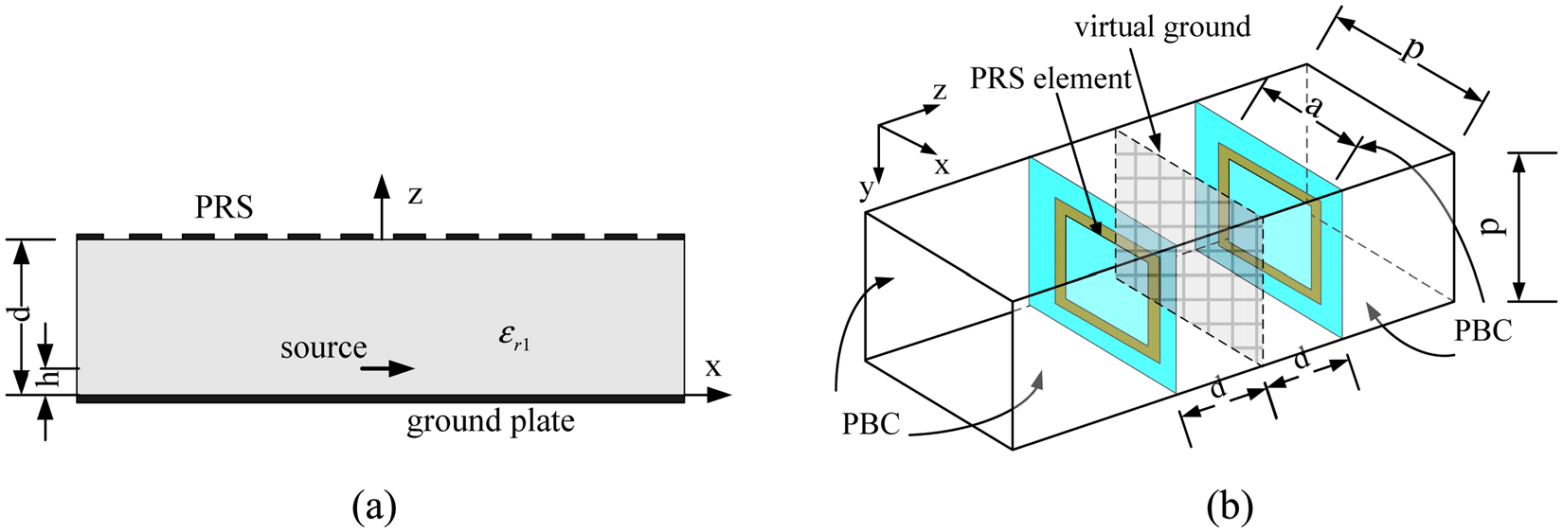
(**a**) Geometry of FPR antenna (**b**) Equivalent cavity model composed of metasurface unit cell and its image.

Figure 1a illustrates the geometry of the proposed FPRA. The resonant cavity is composed of a completely reflecting surface PEC, a PRS formed by metasurface array printed on the bottom surface of a dielectric substrate with permittivity *ε*_*r*1_(*ε*_*r*1_ can also be equal to 1, which means air substrate). The distance between two parallel planes is *d*. An x-directional primary source placed at a distance *h* from the ground plane. As stated before, the interaction between PRS and ground plate can’t be ignored and separated completely, it need to be considered carefully. Thus, the whole FP cavity can be behaved as a homogenous material from the effective medium point of view, when the periodicity of PRS element is much less than the wavelength. Then the equivalent refractive index of the entire structure can be obtained by S-parameter of two-port network retrieval techniques. For FP cavity structure, in order to extract the constitutive parameter, the image theory is utilized in which the ground plane is removed and the virtually mirrored image of the PRS element is copied on the opposite side of the virtual ground plane, as shown in Fig. 1b. The geometry of metasurface unitcell is square ring. The periodic boundary condition is applied to the four sides of the unitcell. Front and back sides are setup as two waveguide ports to allow electromagnetic wave entering from one port and going out from the other port. This model can be used to estimate the transmission and reflection of the whole structure, under the normal incidence. Full-wave simulations using Ansys HFSS are performed to numerically simulate. For the parameter retrieval, the relationship between a S-parameter and a refractive index and impedance has been used, which is written by formula (1–3)^20, 21^, where *n*_eff_ denotes an effective refractive index, *k*_0_ is the wave number in free space, Z is impedance normalized by free space wave impedance, and *m* is an integer^20, 21^.

**Figure 2:**
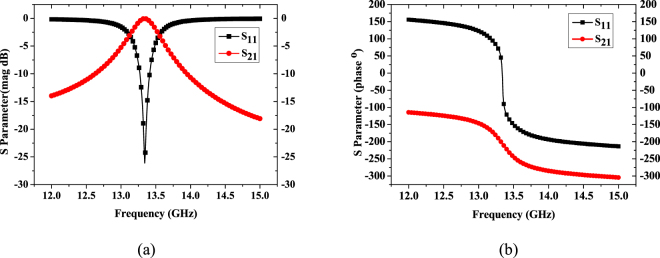
Scattering Parameter of equivalent cavity model (**a**) magnitude (**b**) phase.

**Figure 3:**
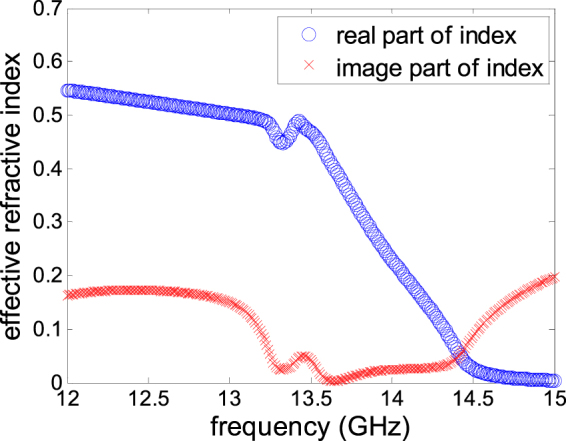
Effective refractive index from equivalent cavity model with *p* = 4.8 mm *a* = 4.4 mm.

For example, the retrieval result of the initially proposed metasurface unitcell with periodicity *p* = 4.8 mm, which is about 1/5–1/6 wavelength level at operating frequency, edge length and width of square ring 4.4 mm and 0.5 mm respectively is shown in Figs 2 and 3. Figure 2 shows the simulated result of frequency response of S-parameter by full wave simulation software HFSS. According to the formula (1–3), we can calculate the effective refractive index by Matlab. As shown in Fig. 3, from 13.6 to 13.7 GHz, the real part of effective refractive index is less than 0.35, while the imaginary part of which in same frequency region is equal to zero. It indicates that the whole resonant cavity can be thought of as a lossless medium with an effectively low refractive index *n*_eff_ in that frequency range.

1 $$Z=\pm \sqrt{\frac{{(1+{S}_{11})}^{2}-{S}_{21}^{2}}{{(1-{S}_{11})}^{2}-{S}_{21}^{2}}},$$

2 $${e}^{j2n{k}_{0}d}=\frac{{S}_{21}}{1-{S}_{11}\frac{Z-1}{Z+1}}.$$

3 $${n}_{eff}=\frac{\text{Im}\{\mathrm{ln}({e}^{j2n{k}_{0}d})\}+2m\pi -j\mathrm{Re}\{\mathrm{ln}({e}^{j2n{k}_{0}d})\}}{2{k}_{0}d}.$$

### Explanation of FPRA with Low Refractive Index

**Figure 4:**
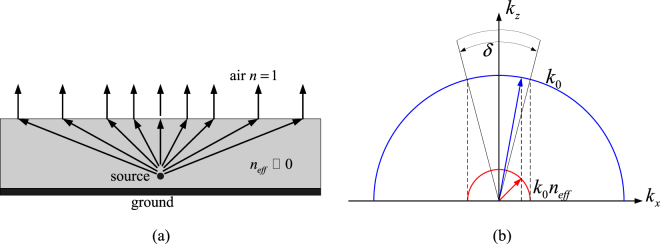
(**a**) Schematic of refractive lens effect (**b**) Graphical construction of dispersion curve.

According to Snell’s law expressed as formula (4), when a source is placed in a medium with a refractive

4 $$\frac{\sin \,{\theta }_{i}}{\sin \,{\theta }_{t}}=\frac{{n}_{t}}{{n}_{i}}.$$

index *n*_i_ near to zero, as shown in Fig. 4a, at the boundary between this medium and free space, we obtain a *θ*_t_ of zero regardless of what *θ*_i_ is. It is indicated that the exiting ray from the top surface of substrate will be normal to the surface, then this property can be used to control the direction of emission and enhance the directivity.

As shown in Fig. 4b, the basic concept that govern the radiation phenomena involved in here can be also illustrated using a graphical construction that satisfies the continuity of the tangential component of the wave vector at the interface between the effective homogeneous medium and the outside background medium^22, 23^. The dispersion curve indicates that the transmitted wave is concentrated within δ angular region. Thus, the whole structure of FP cavity can be acted as an effective metamaterial media with very low refractive index^5^.

### Broaden bandwidth technique

The cavity resonance, though increasing the directivity of simple radiating sources, narrows the operational bandwidth significantly. As shown in Fig. 3, the bandwidth of low refractive index is very narrow. Then the high directive emission enhancement and the bandwidth broadening simultaneously become a severe challenge. One of the simplest methods is to decrease the Q-factor by reducing the module of reflective coefficient of the cover^24, 25^, however, the cost is decreasing the directivity and aperture efficiency. A double-layer metasurface with the same periodicity with different alignments corresponding to slightly different resonate frequencies^26, 27^ can extend the bandwidth, however, the resulting peak directivity and aperture efficiency were deficient. Another method to achieve broadband operation is replace the simple feed by the array^28, 29^, which making the field distribution of the aperture more uniform and hence attaining a wide bandwidth of gain, but a complex feeding network becomes a prerequisite. Recently, PRS with positive reflection phase approach^30, 31^ are also proposed to enhance the bandwidth of FPRA. Furthermore, methods of using a truncated dielectric slab with quarter-wavelength thickness or half-wavelength thickness^32–35^ have been reported to obtain wideband operation. However, a dielectric substrate with a thickness of around quarter- or half wavelength may not be commercially available and they may not be feasible in some circumstances, especially in the low frequency bands.

**Figure 5:**
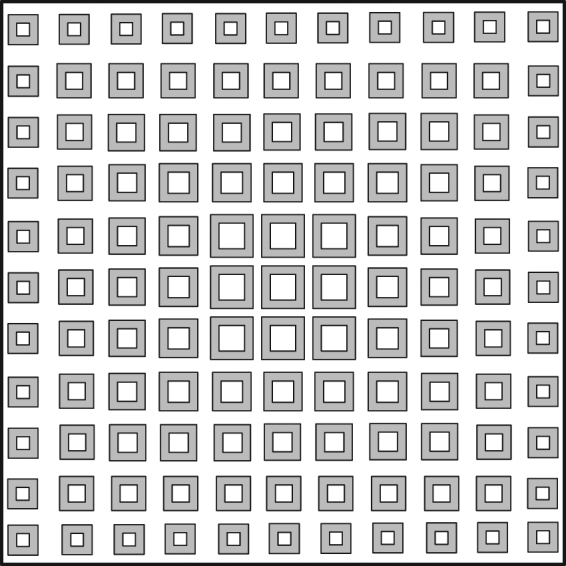
Structure of tapered Metasurface.

**Figure 6:**
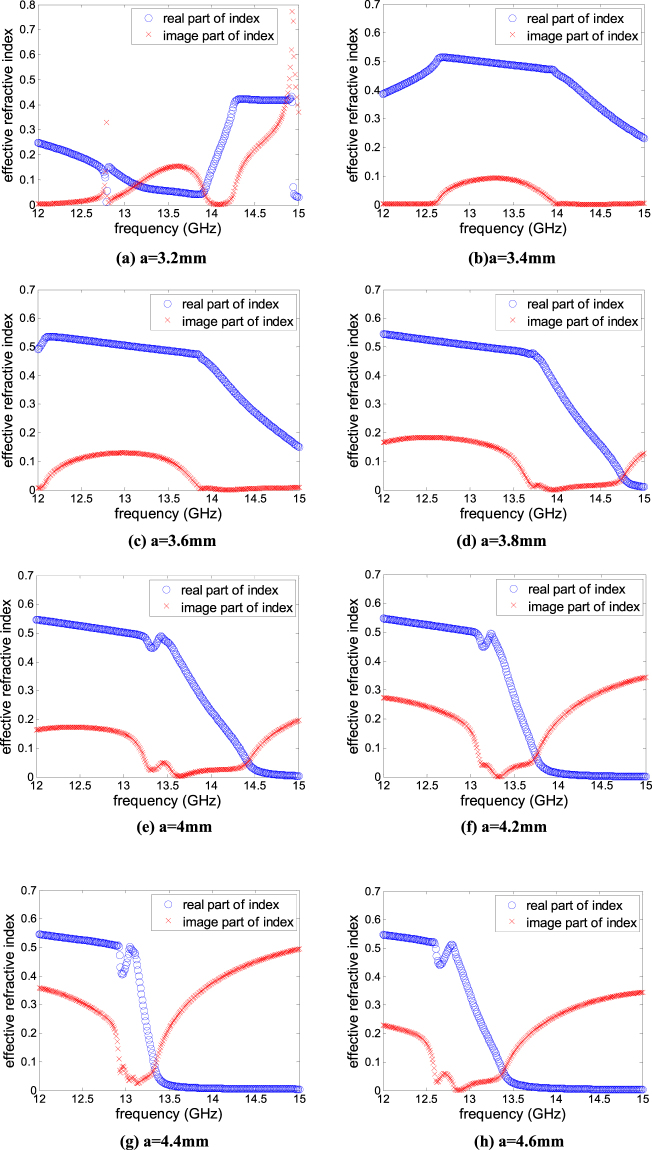
Effective refractive index from equivalent cavity model with different a.

In this paper, the broadband FPRA with high directive emission is implemented by using tapered metasurface structures printed on the bottom surface of thin dielectric slab to form PRS inspired from author previous work^36^. It can be created by gradually modifying the lattice periodicity or the element size of a uniform metasurface structure. Therefore, the effective refractive index can be manipulated by tuning the scale of square ring element. As shown in Fig. 5, when the edge length of square ring is equal to a = 3.2 mm, the frequency range in which the real part of effective refractive index is much less than 1, and the imaginary part of index is very near to zero is from 12 GHz to 12.4 GHz and from 13.95 GHz to 14.15 GHz. As increase edge size of metasurface element, the downward shifting of bandwidth corresponding to the lower refractive index is found, as shown in Fig. 6. Following this principle, by fabricating different scale of ring elements on the same superstrate, their frequency corresponding to the lower refractive index (the real part of effective refractive index is much less than 1, and the imaginary part of index is very near to zero) can be overlapped and therefore, the whole bandwidth yielding high directivity are to be widened. The distinct advantage of our method is the absence of any complexity compared with the previously mentioned method.

### Validation design

**Figure 7:**
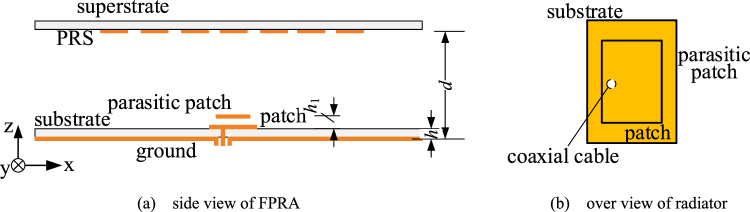
Structure of proposed FPRA and stacked rectangular patch radiator.

**Figure 8:**
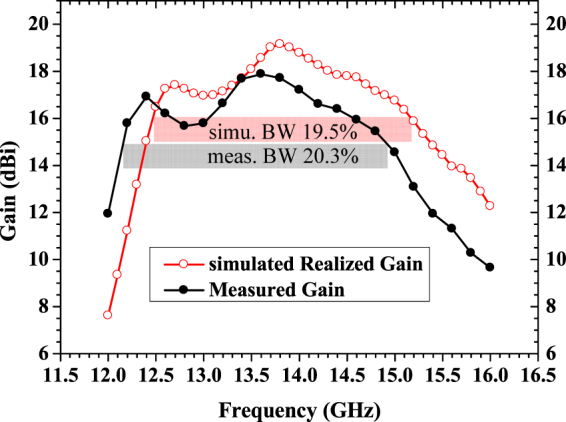
Simulated and measured results of directivity vs frequency for proposed FPRA with tapered metasurface to form PRS.

**Figure 9:**
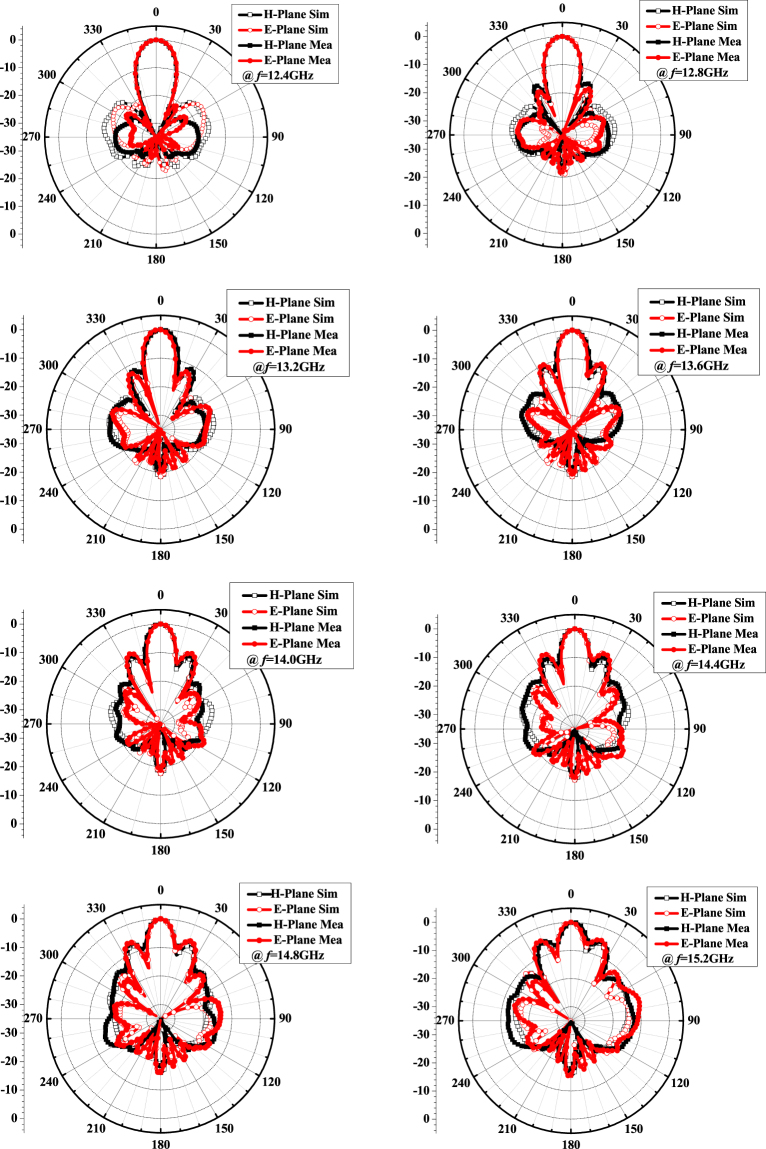
Simulated and measured far field distribution of the proposed FPRA with tapered metasurfaces to form PRS in the E- and H- plane at 12.4 GHz, 12.8 GHz, 13.2 GHz,13.6 GHz, 14.0 GHz, 14.4 GHz, 14.8 GHz and 15.2 GHz.

In order to validate the proposed idea, a broad band FRRA with high directive emission by tapered metasurface is design. The side view of proposed FPRA is shown in Fig. 7a. As shown in Fig. 7b, to enhance the bandwidth of impedance matching, a probe-fed rectangular patch radiator with stack parasitic patch is employed as the feed of FPRA, which is printed on the dielectric sheet with relative permittivity *ε*_r_ = 2.2 and thickness *h* = 1.575 mm. The distance between the radiator patch and stack parasitic patch *h*_1_ is 1.4 mm. The tapered metasurfaces are printed on the bottom surface of superstrate with permittivity *ε*_r_ = 2.2 and thickness *h* = 1.575 mm. The height of FP cavity *d* is 12.5 mm, which is around a half wavelength of operating frequency. The optimized simulation results of frequency response of gain are shown in Fig. 8. The simulated peak realized gain approaches 19.1 dBi, and 19.5% bandwidth of 3-dB gain drop (12.4~15.2 GHz) is also obtained. Far-field reports of the radiation pattern are also presented in Fig. 9 over a broad frequency range. In simulation, we did not spend much time to optimize the arrangement of rings. It is possible to optimize them to boost the performance further.

**Figure 10:**
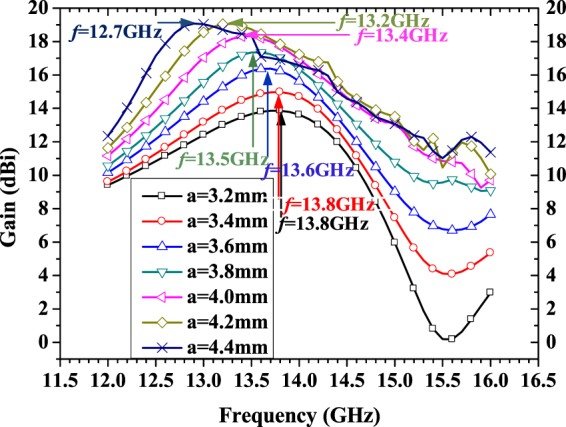
Comparison of directivity vs frequency for FPRA with different size of metasurface unitcell to form uniform PRS.

In order to explain clearly, the gain frequency response of FPRA with uniform metasurfaces to form PRS are compared in Fig. 10. In fact due to the very narrow gap between adjacent elements could happen when using a = 4.6 mm.Then in Fig. 10, the plot for a = 4.6 mm is missing. It is illustrated that, when implementing different size of metasurfaces unitcell to form the uniform PRSs, the frequency corresponding to peak gain of FPRA is coincide with the frequency in which the real part of effective refractive index is much less than 1, and the imaginary part of index is very near to zero as plotted in Fig. 6. It is worth noted that there exist shifts between these two frequency response, when a = 3.2 mm and a = 4.4 mm. The main reason is that the effective refractive index as shown in Fig. 6 retrieved from model as shown in Fig. 1 reveals the constitutive parameter of the model itself, the excitation factor is not included. But Fig. 10 compared the gain vs frequency for FPRA with different size of metasurface unitcell, it means that the practical excitation is involved. So the frequency corresponding to peak gain in Fig. 10 may have little shift with frequency in which real part of effective refractive index is less than 1 and the imaginary part is near to zero. Although, it is indicated that the mechanism of high directive emission of FPRA explained by effective refractive index near to zero and its retrieval method mentioned above is correct and feasible. At same time, we also observed that the directivity bandwidth of FPRA when implementing uniform metasurfaces are narrow. And the values of bandwidth of 3-dB gain-drop, peak gain and center frequency for different cases of FPRA are presented in Table 1. In addition, a comparison of antenna characteristics between the proposed antenna and the previous studies^6–8, 17, 31, 32^ is shown in Table 2.

**Table 1:** Performance comparison of different cases of FPRA.

Size of MES (mm)	a = 3.2	a = 3.4	a = 3.6	a = 3.8	a = 4.0	a = 4.2	a = 4.4
Center fre. (GHz)	13.8	13.8	13.6	13.5	13.4	13.2	12.7
Peak gain (dBi)	13.86	14.97	16.37	17.35	18.31	19.05	19.07
Bandwidth (GHz / %)	2/14.4	1.8/13.0	1.6/11.8	1.9/14.1	1.6/11.9	1.7/12.9	1.8/14.2

**Table 2:** Performance comparison of some previous work.

Configuration	oversize	Peak gain, dBi	3 dB gain BW,%
[6]	3.14 * 1.66 * 1.66 * 3.15 ${\lambda }_{0}^{3}$	23.1	9.2–12.6 GHz, 31.2
[7]	3.14 * 2.94 * 2.94 * 4.9 ${\lambda }_{0}^{3}$	22.5	5.5–7.0 GHz, 24
[8]	1.63 * 1.57 * 1.5 ${\lambda }_{0}^{3}$	15.5	4.0–6.0 GHz, 40
[17]	1.33 * 1.33 * 0.17 ${\lambda }_{0}^{3}$	9.5	—
[31]	11.8 * 11.8 * 0.6 ${\lambda }_{0}^{3}$	16.2	11–13 GHz, 15.7
[32]	2.8 * 2.8 * 1.3 ${\lambda }_{0}^{3}$	18.7	11–13.4 GHz, 19.6
proposed	3.2 * 3.2 * 0.6 ${\lambda }_{0}^{3}$	17.9	12.2–14.9 GHz, 20.3

## Method

### Fabrication and Experiment

**Figure 11:**
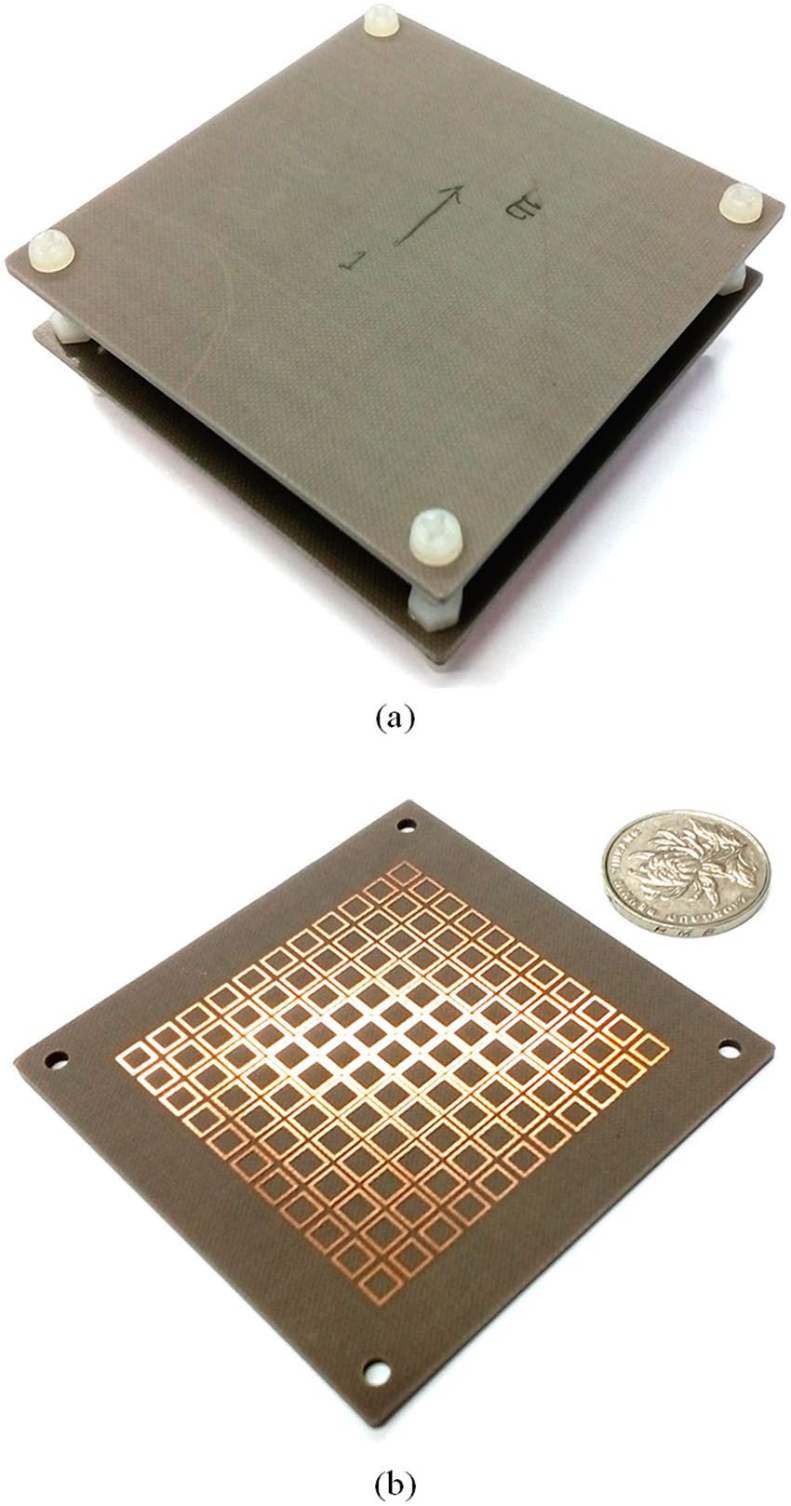
Prototype photo of proposed FPRA with tapered metasurfaces to form PRS. (**a**) solid view, (**b**) PRS with tapered metasurfaces.

In order to validate the simulation results, as shown in Fig. 11, a prototype with standard PCB technology is fabricated for testing. The high directive emission features of the fabricated sample are measured in chamber. As shown in Fig. 8, compared with simulation results, the measured 3-dB gain bandwidth of 20.3% from 12.2 GHz to 14.9 GHz is obtained, which is a little wider than the simulated one. There is about 0.2 GHz frequency downward shift. The maximum measured gain is about 17.9 dBi at 13.6 GHz, which has a 1.2 dB drop from the simulated result. A good agreement between simulated and measured results confirm that the approach to enhance the bandwidth and to possess a high directive emission of FPRA simultaneously by tapered metasurfaces is effective.

## Conclusion

In this paper, we bring novel explanation of near-zero effective index on high directive emission of Fabry-Perot resonator antenna with subwavelength metasurface and also present a good scheme with tapered near-zero effective index using metasurface to improve the bandwidth and high directive emission simultaneously of such antenna. In addition, the new method for retrieval of the constitutive parameter of metasurfaces when the distance between source and metasurface sheet do not meet the far field condition. As an example, a final prototype has been fabricated and measured to validate the proposed design concept. The measured 3-dB gain bandwidth is enhanced to approach 20.3% with a peak gain of 17.9 dBi.
